# Evaluation of the Leap Motion Controller as a New Contact-Free Pointing Device

**DOI:** 10.3390/s150100214

**Published:** 2014-12-24

**Authors:** Daniel Bachmann, Frank Weichert, Gerhard Rinkenauer

**Affiliations:** 1 Department of Computer Science VII, Technical University Dortmund, Dortmund 44221, Germany; E-Mails: daniel.bachmann@uni-dortmund.de (D.B.); frank.weichert@tu-dortmund.de (F.W.); 2 Leibniz Research Centre for Working Environment and Human Factors, Dortmund 44139, Germany

**Keywords:** Leap Motion Controller, Fitts' law, human-computer interaction

## Abstract

This paper presents a Fitts' law-based analysis of the user's performance in selection tasks with the Leap Motion Controller compared with a standard mouse device. The Leap Motion Controller (LMC) is a new contact-free input system for gesture-based human-computer interaction with declared sub-millimeter accuracy. Up to this point, there has hardly been any systematic evaluation of this new system available. With an error rate of 7.8 % for the LMC and 2.8% for the mouse device, movement times twice as large as for a mouse device and high overall effort ratings, the Leap Motion Controller's performance as an input device for everyday generic computer pointing tasks is rather limited, at least with regard to the selection recognition provided by the LMC.

## 1. Introduction

Effective, efficient human-computer interaction (HCI) is strongly influenced by input devices, which sense the physical interaction of users. There is a large variety of classical input devices, e.g., mouse, trackball, joystick, touch pad or touch screen. Besides the classical input devices, there are more and more contact-free input systems available, e.g., gesture-recognition [1], eye-gaze control [2] or speech input [3]. Such sensors become significant for sterile environments, e.g., medical or industrial applications [4]. Different input device characteristics, however, make different requirements of human abilities. Therefore, the compatibility between device characteristics and the abilities of the user determines the objective and subjective input performance to a large extent.

This paper evaluates the Leap Motion Controller (LMC) as a new three-dimensional (3D) contact-free pointing device. The Leap Motion Controller introduces a new gesture and position tracking system with sub-millimeter accuracy. In contrast to standard multi-touch solutions, this touch-free sensor is discussed for use in realistic stereo 3D interaction systems [5], especially concerning the direct selection of stereoscopically-displayed objects [6]. Further, exploration of the suitability of the Leap Motion Controller for Australian Sign Language (Auslan) [7] and handwriting recognition [8] are discussed. Design issues and opportunities for bare-hand 3D controlling techniques with the LMC are evaluated [9]. The performance of a mouse device and the LMC for 3D manipulation tasks is analyzed [10], and an LMC-supported Augmented Reality (AR) interface is proposed [11]. Evaluations of a device as a pointing device is according to the International Organization for Standardization (ISO) 9241-9:2000 standard [12]. The norm depicts the necessary issues to calculate the device performance (throughput). This is performed in different studies, e.g., [13–23]. Six mouse devices with small variations in size and shape are analyzed without significant performance differences [14]. An isometric joystick and a touchpad are evaluated as pointing devices, finding non-significant differences for one-dimensional tasks and 27% higher throughput for the joystick in two-dimensional tasks [15]. Different designs for isometric joysticks are discussed and evaluated for handheld information terminals [16]. Pino *et al.* [17] are evaluating the performance of computer-based pointing tasks for a Microsoft Kinect device and a mouse. In 2D pointing tasks, throughput is almost 39% lower than using the mouse, and the missed clicks count is almost 50% higher. New devices, including the Wii Remote, are discussed as devices for the manipulation of 3D scenes [18,19]. A classical game controller and the Wii Remote are evaluated using a mouse as a baseline condition [20]. The evaluation of a standard game controller and a modified trackball game controller is presented by Natapov and MacKenzie [22], showing a performance gain of 60% compared to the standard device. Three pointing devices for remote pointing are evaluated for applicability to interactive TV environments and compared to a standard mouse device [21] with poor performance compared to the baseline condition, and a computer-controlled laser pointer is discussed as a pointing device in a collaborative environment. Results show approximately 75% of the performance of a standard mouse device [23]. Bérard *et al.* [19] found superior performance for a standard mouse device in the accuracy of 3D positioning tasks, while Balakrishnan *et al.* [18] propose a modified mouse device that is 30% faster than a standard mouse in a custom-designed positioning task. In general, an improvement of human-computer interfaces is necessary in view of the increased complexity of computer-based systems [24]. Especially, touch-free devices, like the Leap Motion Controller, will now also be important, e.g., to prevent contamination from pathogens in medical and biological workplaces [25] or as an interaction device in telemedicine [26].

To the knowledge of the authors, the user performance with the Leap Motion Controller has not been systematically analyzed yet.

One important aspect of computer input research is to measure and characterize the performance of various input systems. Given the great potential diversity of input devices, as mentioned above, a critical need is to be able to compare and characterize them from a human performance perspective. Numerous researchers have used concepts from information theory to describe interactions with information technology. Fitts' law is arguably the most widely-known example [27–29] and is frequently used to compare the human performance with input devices. It describes the relationship between movement time, distance and accuracy in rapid aimed movements [30] and, thus, applies to the pointing and dragging interactions of a wide variety of standard devices, like a mouse, trackball, stylus, *etc.*, as well as of contact-free devices.

It implies a tradeoff between speed and accuracy, suggesting that movement time increases as navigation distance increases or the size of the target decreases [30]. In the context of Fitts' law, each attempt to select a target is viewed as being a success or failure. Selection time and error rate are used to describe the efficacy of interaction solutions.

This paper presents a study of the Leap Motion Controller's abilities as a pointing device. To compare pointing tasks performed with the two devices, participants were instructed to execute pointing and click interactions as they are provided by the two devices. Therefore, in the mouse movement conditions, the cursor had to be moved to the actually labeled target and the selection had to be performed by a click. Analogously, in the LMC movement conditions, the cursor also had to be moved to the actually labeled target by the index finger and selection had to be performed by a quick vertical finger movement. However, the latter gesture, which is supposed to be an analogy of the mouse click, is more difficult to control than a mouse click and, thus, may cut down the performance of the LMC device. The main focus of this paper therefore is on:

- Performance: Evaluation of the performance differences between mouse and the LMC for click and pointing gestures. More degrees of freedom have to be controlled for the Leap Motion Controller compared to the mouse. Furthermore, the execution of pointing movements with the LMC is more complex. Thus it is hypothesized that the performance may suffer from the additional load.
- Practice: Given that there are performance differences between mouse and LMC, it is evaluated if practice may help to overcome such differences.
- Gender: Rohr [31] found some effects for gender-specific movement strategies in the context of a computer-pointing task. Therefore, this study also aims to control for gender effects.

The aim of this study is to provide an experimental approach that focuses on the main axis (horizontal axis) of interaction only. Horizontal movements are required independently of the interaction device (e.g., mouse, touch pad, touch screen) and the manipulation plane (horizontally *vs.* vertically). Furthermore, additionally to the performance analyzes provided by Fitts' task, it is discussed to what extent movement trajectory characteristics are affected by different levels of task difficulty. The analyses of the trajectories may especially help to investigate the click gesture of the LMC in more detail. For such analyses, repeated movements for each of the employed combination of distances and target widths are needed. Therefore, the ISO 9241-9:2000 standard task is not adopted yet, because in the instructed procedure of this task, the direction of the subsequent movements has to be changed. Movements in different directions, however, require different muscle groups and different nonlinear transformations of the movements around the joints for the different 2D movement directions. Thus, the trajectories conducted in the ISO standard task may not be comparable within a certain level of difficulty, and the analyses of the LMC movements may be confounded by movement direction.

The paper is organized as follows. In Section 2, the materials and methods are introduced. First, the LMC is explained, and the framework of Fitts is presented in Section 2.1. Section 2.2 to Section 2.5 expose the participants and the experimental set-up in detail. Results are presented in Section 3, and the Discussion and Conclusion are conducted in Section 4.

## 2. Materials and Methods

**Figure 1.**
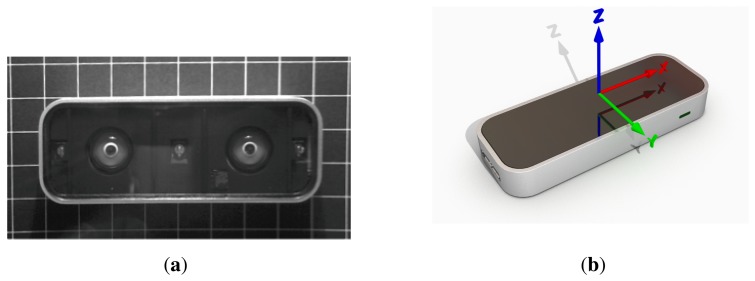
Visualization of a (**a**) real (using infrared imaging) and (**b**) 3D model of the Leap Motion Controller with the coordinate system.

The Leap Motion Controller (LMC) is a new consumer-grade sensor developed by Leap Motion (Leap Motion, http://www.leapmotion.com). It is primarily designed for hand gesture and finger position detection in interactive software applications. Up to this point, only insufficient information on the underlying software's geometrical or mathematical frameworks has been available. Figure 1a shows an infra-red image of the controller's hardware setup. Besides three infrared emitters, the device incorporates two IR cameras. As stated by the manufacturer, the sensors accuracy in position detection is about 0.01 mm. Recent research [32] has shown that an accuracy of below 0.2 mm for static setups and of 0.4 mm for dynamic setups was obtained in realistic scenarios. The precision and reliability of the LMC were analyzed for static and dynamic scenarios using a high-precision optical tracking system [33] as the ground truth. A standard deviation of less than 0.5 mm for static scenarios and an inconsistent performance for dynamic scenarios were obtained. This shows a high potential of the LMC for gesture-detection systems in HCI applications. However, the limiting factor for the obtainable user performance in pointing tasks is not the accuracy and precision of the LMC. For static setups, the LMC's accuracy is below the human hand tremor [34,35]. This implies that the main restriction for effective pointing task performance with the LMC is, besides the complexity of the gesture used to perform a click movement, the human motor system itself.

Hand and fingertip positions are detected in coordinates relative to the center of the controller in a right-handed coordinate system (*cf.* Figure 1b). Further, different types of gestures are detectable (Leap Motion API, http://developer.leapmotion.com/documentation/cpp/devguide/Leap_Overview.html). Direct access of raw IR images was not provided via the utilized LMC software developer kit (Version 0.7.9). The so-called tap gesture was chosen as the pointing movement equal to a mouse click. The tap gesture was recognized if the fingertip rotated down towards the palm and afterwards back to its original position with a velocity of at least 40 mm/s.

### 2.1. Fitts' Law as a Theoretical Framework to Assess User Performance

As mentioned in the Introduction, Fitts' law probably is the most frequently-used theoretical framework to describe and compare user performance for different input devices. On the one hand, the framework is used as a predictive model, *viz.* to predict the time to move the cursor to a button and click on it. On the other hand, it is used as a means to estimate the throughput as an index of performance of the human motor system. Fitts' law actually applies information theory to human behavior, and its basic assumption is that human performance can be described with the mathematical concept of “information” as, for example, used in electronic communication systems [36]. The amplitudes of aiming movements are considered in analogy to the information of signals transmitted in electronic systems and the variability (spatial accuracy) of the movements in analogy to electronic noise. Furthermore, it is assumed that the human motor system is like a communications channel, where movements are like the transmission of signals and the bandwidth (information capacity) of the channel can be expressed in bits/s.

User performance in the current study was assessed in conformity to Fitts' law [27]. Thus, task difficulty was quantified as the index of difficulty (*ID* in bits) using information theory. More specifically, the Shannon formulation, the widely-used version of the index of difficulty (*ID*) [28,37]:

(1) $$ID=\log_{2}(\frac{D}{W}+1)$$

was utilized, where *D* is the distance between targets and *W* is the target width. The units of *ID* is bits and emerges from the base 2 of the logarithm. *ID* was used in this paper as the factor to manipulate task difficulty. As a main dependent variable, movement time (*MT*) was used to assess task performance. Fitts' law holds that *MT* is a linear function of *ID*, characterizing a movement along a single dimension:

(2) MT=a+bID

Fitts also proposed to quantify the human rate of information processing in aimed movements. The index of performance, called throughput (*TP*), is calculated by dividing the effective index of difficulty *ID_e_* by *MT*, computed over a block of trials:

(3) $$TP=\frac{ID_{e}}{MT}$$

The units of *TP* are bits per second (bps). *ID_e_* is used because endpoints of target selections have much lower variance, as one might expect [38], and is defined as:

(4) $$ID_{e}=\log_{2}(\frac{D}{W_{e}}+1)$$

Here, *W_e_* is the effective target width computed from the variability of the observed endpoints:

(5) $$W_{e}=4.133\cdot SD_{x}$$

where *SD_x_* is the standard deviation in the selection coordinates measured along the axis of approach to the target. Because of the chosen LMC pointing gesture (tap gesture), which makes it difficult to robustly and precisely determine the end of the pointing movement and beginning of the movement to the next target, the effective distance *D_e_* was not used in the formulation of the effective index of difficulty, as proposed by Soukoreff and MacKenzie [37].

### 2.2. Participants

Twelve (6 male) right-handed undergraduates participated in a 1.5-h session (mean age = 25.25 years, SD = 4.22 years). All participants had corrected-to-normal vision and used computers with modern GUIs and a mouse on a daily basis. None had prior experience with the LMC. All participants used their preferred hand during the experiment.

### 2.3. Apparatus

**Figure 2.**
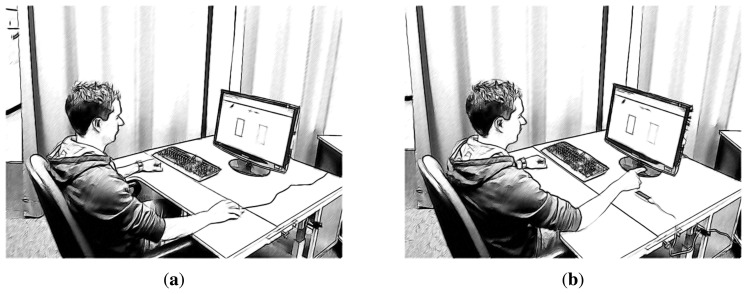
Experimental set-up: (**a**) participant using the mouse controller and (**b**) utilizing the Leap Motion Controller (LMC) to perform selection tasks.

A custom-designed software, written in C++, was used to present the different tasks and to record the participant's movement trajectories. The graphical interface was designed using the Qt-Framework (Qt Project, http://www.qt-project.org) and presented on a 22-in TFT, which was running at a resolution of 1680×1280 pixels. The devices consisted of a Logitech optical mouse (Model RX 250 (Logitech RX250 Mouse, http://www.logitech.com/de-de/product/rx250-optical-mouse-business) and the LMC. The user interface was adapted to the screen's resolution, such that a mapping from LMC coordinates (mm) to screen coordinates (pixels) was realized. Movement acceleration of the mouse was matched to the horizontal range of the LMC, such that a horizontal movement of approximately 300 mm regarding both devices (position of the mouse controller and the fingertip position over LMC) was required to cover the screen's horizontal extent. The screen surface was oriented vertically in front of the participants with a distance of 70 cm. Mouse movements were performed on the right side below the screen's surface, on the table at which the participants were sitting. The LMC was also placed at the right side of the screen, such that the horizontal movement range was identical to the movement range of the mouse (*cf.* Figure 2). To avoid displacement, the LMC was fixed to the table's surface with adhesive tape. The height of the chair and the distance between chair and table were adjusted by the participants to a comfortable height. To reduce strain during the interaction with the LMC, participants were instructed to put their elbow on the table (see Figure 2b).

### 2.4. Design

Data analysis was based on a 2 (device) × 2 (gender) × 5 (*ID*) × 3 (block) mixed design. The design consisted of the between-group factor gender and the within-group factors device (mouse *vs.* LMC), index of difficulty (*ID* ∈ {1, 1.6, 2.3, 3.2, 4.1}) and block (1, 2, 3;. With regard to the factor device, participants performed in the mouse condition pointing movements using a mouse in the conventional manner and selecting the target by a click of the left mouse button. In the LMC condition, participants used their index finger to interact with the LMC device (*cf.* Figure 2b). In the LMC condition, participants had to perform aiming movements between the targets and select the respective destination targets by an additional pointing movement (tap gesture). The different *ID* conditions were combinations of varying distances and target widths (see Table 1). To assess training and learning effects, each combination of the factors device and the index of difficulty was performed in each of the three consecutive blocks. Each participant received a different order of the factors target widths, target height and device, based on a Latin square. For each of the conditions, 20 selections were performed. To measure and compare the performance of the two devices, three dependent variables were used: movement time (*MT* in ms), error rate (*ER* in %) and throughput (*TP* in bps). *MT* was the mean time per trial, including both the time to move from the source target to the respective destination target and the time to make a selection. The error rate was the percentage of out-of-target selections. *TP* was calculated according to Equation (3) (*cf.* Section 2.1).

### 2.5. Procedures

**Figure 3.**
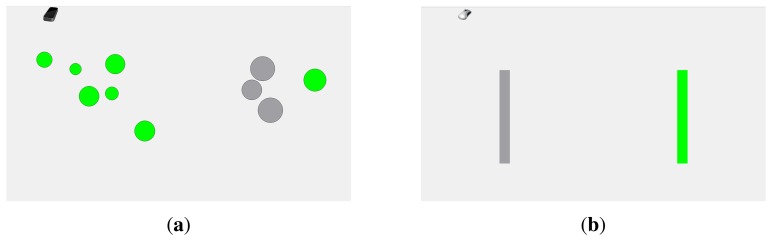
Scaled illustrations of the graphical user interface. Selected targets are displayed in gray; selectable targets are colored green: (**a**) the additional custom-designed training phase for the LMC pointing task; and (**b**) the interface for the pointing task.

Prior to testing, the participants were briefed on the purpose of the experiment. Tasks were explained and demonstrated, and participants were advised to move quickly while minimizing errors. Because none of the participants was familiar with the LMC, a short custom-designed training was provided at the beginning of a session. The instructed training task was different from the experimental task in order to avoid the practice advantages of the LMC over the mouse device. In order to practice the pointing gesture with the LMC, a set of 10 randomly arranged circular targets of random radii between 7 and 20 mm were displayed on the screen (*cf.* Figure 3a). The training was completed after all targets were selected by performing the pointing gesture, while aiming at a point inside one of the not already selected circular targets. The completion of a selection was indicated by a change in the target's color. After the specific LMC training, the experimental task for both devices was practiced with the two devices. During this second practice phase, participants had to perform one serial selection task for each distance/width condition with the mouse, as well as with the LMC device.

After the practice phase, the experimental blocks were started. At the beginning of each block, a screen was displayed indicating which device was tested next. For easier detection of device changes, the upcoming device was displayed as a picture in the upper left part of the screen (see Figure 3). The start of a new trial was triggered at the participant's discretion. In each trial, two targets of width *W*, separated by distance *D*, were displayed on the screen. Randomly, one of the targets was annotated as the starting target, highlighted in green. The task was to alternately select the targets in the implied order by performing a device-specific click. According to Fitts' paradigm [27], this complies with the so-called serial selection task. The cursor was initially set to the center position between the targets, *i.e.*, the serial selection task is initialized by one discrete task, which was not included in the data analysis. After the cursor's starting position changed by more than 5 mm, the recording of the movement trajectories was activated. After a successful selection, the selected target's color changed to gray. Missing the target (performing a click outside the target area) or selecting the wrong target was emphasized by highlighting the missed target. For each distance/width condition, 10 back-and-forth selections were performed.

After each block, participants were asked to rate the subjective difficulty of the tasks. For these ratings, the rating scale of mental effort (RSME) [39] was employed.

## 3. Results

Based on the framework of Fitts (*cf.* Section 2.1), the observed user performance was evaluated. In Sections 3.1 to 3.3, the error rate, movement time and throughput are presented. The mental effort is evaluated in Section 3.4. Data were submitted to an analysis of variance (ANOVA) [40,41]. The probability level for the statistical significance of all analyses was *p* < 0.05. Greenhouse-Geisser-corrected results are reported when necessary [42]. The general eta-squared $\eta_{G}^{2}$ [43] is reported as a measure of the effect size for all significant ANOVA results. Afterwards, accumulator plots are introduced, which represent intuitive visualizations for different patterns of movement trajectories.

### 3.1. Error Rate

**Figure 4.**
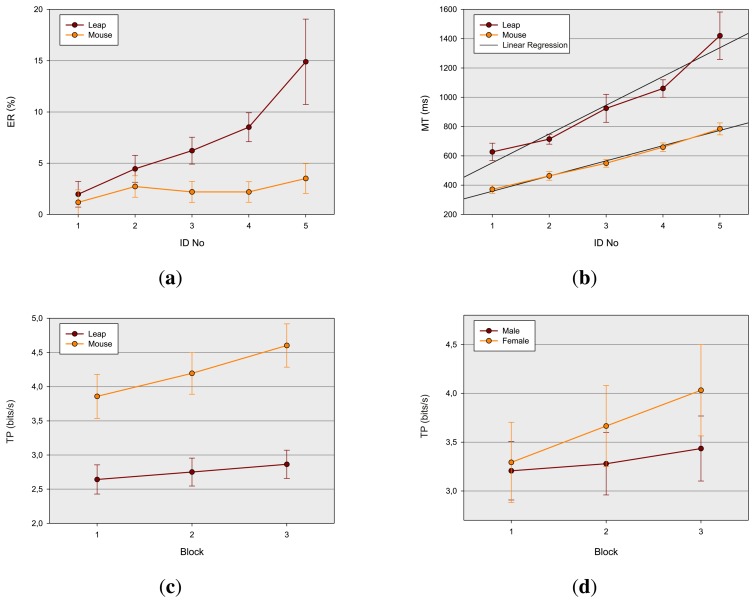
Results of the experiment: (**a**) error rate (*ER*) by device and index of difficulty (*ID*); (**b**) movement time (*MT*) by device and *ID* with linear regression lines fitted to the data; (**c**) throughput (*TP*) by block and device; and (**d**) *TP* by block and gender. Error bars indicate the 95-percent confidence intervals.

The mean error rate over the entire experiment was 4.8 %. The error rate was about three-times higher when movements were performed with the LMC (7.2 %) than with the mouse (2.3%), F(1, 10) = 41.6, *p* < 0.001, $\eta_{G}^{2}=0.19$. As expected, the error rate increased with *ID* and was lowest (1.6 %) at *ID* = 1 and highest (9.2 %) at *ID* = 4.1, F(4, 60) = 21.3, *p* < 0.001, $\eta_{G}^{2}=0.2$. There was a steeper increase of the error rate with *ID* for LMC movements than for mouse movements, F(4, 40) = 10.0, *p* < 0.001, $\eta_{G}^{2}=0.1$. The different performance patterns between the two devices are depicted in Figure 4a. There was no effect of gender and block, *p*'s > 0.27.

### 3.2. Movement Time

Mean *MT* over the entire experiment was 757 ms. There was a main effect of block, F(2, 20) = 5.9, *p* < 0.02, $\eta_{G}^{2}=0.06$, *viz.* the mean *MT* systematically decreased across the three blocks (817,756,698 ms), suggesting a general practice effect. Movements performed with the LMC lasted considerably longer than mouse movements (945, *vs.* 565 ms), F(1, 10) = 121.8, *p* < 0.001, $\eta_{G}^{2}=0.48$. As expected, *MT* increased with *ID* and was lowest (499 ms) at *ID* = 1 and highest (1102 ms) at *ID* = 4.1, F(4,40) = 95.1, *p* < 0.001, $\eta_{G}^{2}=0.53$. Analogous to the error rates, there was a steeper increase in *MT* for LMC movements than for mouse movements, F(4, 40) = 14.0, *p* < 0.01, $\eta_{G}^{2}=0.11$(see Figure 4b). Again, there was no effect of gender, *p*'s > 0.34. Linear regression shows, that Fitts' law (*cf.* Equation (2)) holds for the mouse device (*a* = 258.52, *b* = 102.26, *r*^2^ = 0.99, standard error of the estimate *e* = 14.44); for the LMC, the predictive qualities of Fitts' law are limited (*a* = 369.12, *b* = 193.28, *r*^2^ = 0.93, *e* = 82.46). The LMC-based trails show less efficiency in target activation than the ones performed with the standard mouse device (369.12 ms *vs.* 258.52 ms) [44].

### 3.3. Throughput

**Figure 5.**
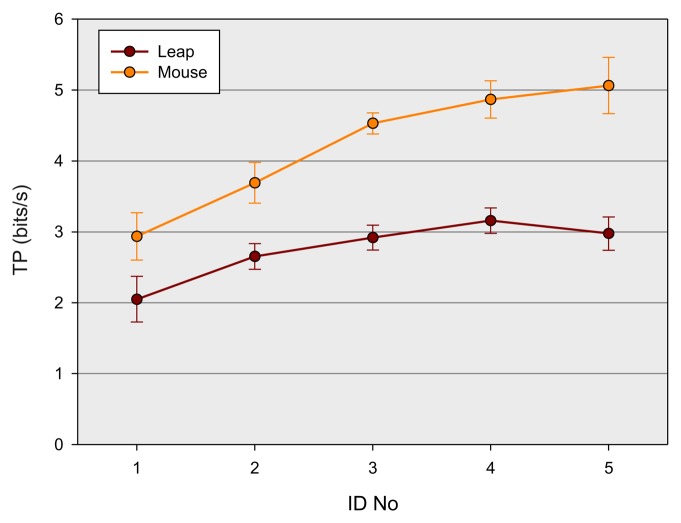
Result of the experiment: throughput (*TP*), the main indicator to estimate the performance of users as a function of device and *ID* number.

As already mentioned earlier in this paper, throughput (*TP*) is an additional important measure of performance, combining both speed and accuracy, and is considered as the main indicator to estimate or predict the performance of users with a certain device. To assess *TP* for the two devices, a three-way ANOVA with the factors gender, block and device was conducted. Mean *TP* over the entire experiment was 3.5 bps. There was a main effect of block, F(2, 20) = 21.0, *p* < 0.001, $\eta_{G}^{2}=0.06$, of device, F(1, 10) = 124.0, *p* < 0.001, $\eta_{G}^{2}=0.45$, but not of gender, *p* > 0.2. The main effect of block indicates that the throughput generally increases significantly with the number of blocks (3.3, 3.5, 3.7 bps). In agreement with the findings for the error rate and the movement time, there was a lower throughput for the LMC than for the mouse movements (2.7 *vs.* 4.2 bps). This main effect was modulated by the factor block, *viz.* there was a steeper growth for the mouse than for the LMC, F(2, 20) = 4.7, *p* = 0.04, $\eta_{G}^{2}=0.017$ with increasing number of blocks (*cf.* Figure 4c). *Post hoc* analyses (Duncan's test [45]) revealed that the *TP* of both devices differed significantly at each level of block (*p* < 0.001). Subsequent tests between each block level within each of the two devices showed that *TP* for the mouse significantly differed between each level of block (*p* < 0.01), which indicates a continuous increase of practice. There were, however, no significant differences for the LMC device between the three levels of block (*p* > 0.09), which suggests that there was no significant practice effect at all. There was also a significant interaction of the factor block and gender F(2, 20) = 5.9, *p* < 0.01, $\eta_{G}^{2}=0.016$ (*cf.* Figure 4d). *Post hoc* tests revealed that *TP* for female participants differed between each level of block (*p* < 0.003), but not for male participants (*p* ≥ 0.06). However, pairwise comparison between the two groups at each level of block failed to reach significance (*p* ≥ 0.10), presumably due to the lack of statistical power of this between-subject comparison. The result, however, gives at least a hint that there was a practice effect for female, but not for male participants. The main effect of block suggests that training provokes a general increase of throughput, independently of the device used. This finding corroborates the general practice effect found for *MT.* In contrast to the earlier studies of Rohr [31], which found lower performance in pointing movements for female than for male participants, no gender-specific training effects on *MT*, but on *TP*, are found. The *TP* of female participants seem to gain more from practice than the *TP* of male participants. No other effects attained significance within the three-way ANOVA (*p* > 0.062). To estimate the maximal possible throughput of the two devices, which could be reached within the range of provided *IDs*, an additional two-way ANOVA with the factors device and *ID* was calculated. There was a main effect of *ID-No*, F(4, 40) = 18.1, *p* < 0.001, $\eta_{G}^{2}=0.34$. As depicted in Figure 5, the throughput of both devices increased differently, F(4, 40) = 11.1, *p* < 0.001, $\eta_{G}^{2}=0.07$. *Post hoc* tests between the two devices revealed that there is a significant difference in *TP* for each level of *ID* (*p* < 0.001). Further *post hoc* comparisons between *ID* levels within each device suggest that *TP* for the mouse device differs significantly (*p* < 0.03) between each *ID* level up to the fourth level. There was no significant difference between *ID* Levels 4 and 5 (*p* = 0.19). For the LMC devise, however, there were subsequent significant differences only between *ID* Levels 1 and 2 (*p* < 0.001) and Level 2 and 4 (*p* < 0.003). There seems to be a drop in *TP* between *ID* Levels 4 and 5, which, however, does not attain statistical significance (*p* = 0.23). The results of the *post hoc* analyses suggest that there is a continuous increase of *TP* for the mouse device, and saturation is reached at *ID* = 3.2 at a maximal *TP* = 5.0 bps (averaged over *ID* Levels 4 and 5). In the LMC device, the increase of *TP* seems to be rather inconsistent in comparison with the mouse device. However, analogous to the mouse device, saturation for *TP* seems also to be reached at *ID* = 3.2 with an average maximum *TP* = 3.1 bps. The findings suggest that the throughput of the LMC device is similarly affected by *ID*, like the mouse device, but at a lower level.

### 3.4. Rating of Mental Effort

For a subjective measure of workload after each track, the rating scale of mental effort (RSME, [39]) was employed. This simple one-dimensional ratio scale allows participants to rate their invested mental effort into a task on a scale from zero (absolutely no effort) to 150 (extreme effort). As already mentioned above, mental effort was rated on the RSME after each experimental block. The mean rating for the LMC was 73.25 (*SD* = 34.83) and 37.64 (*SD* = 29.36) for the mouse device. There was only a main effect of the factor device, *viz.* the use of the LMC was generally rated as more demanding than the use of the mouse, F(1, 10) = 15.6, *p* < 0.01, $\eta_{G}^{2}=0.25$.

**Figure 6.**
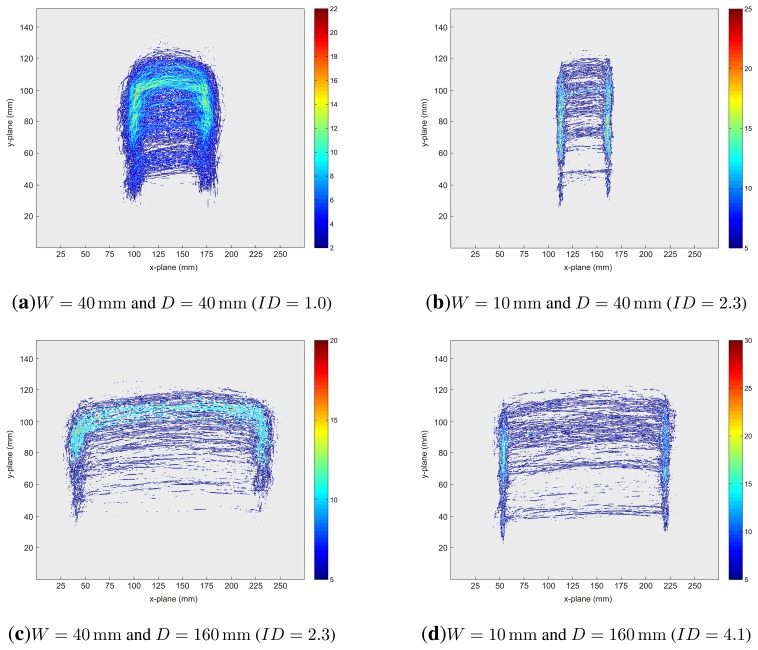
Aggregate accumulator plots for different indices of difficulty: (**a**) *ID* = 1.0, (**b,c**) *ID* = 2.3 and (**d**) *ID* = 4.1.

**Figure 7.**
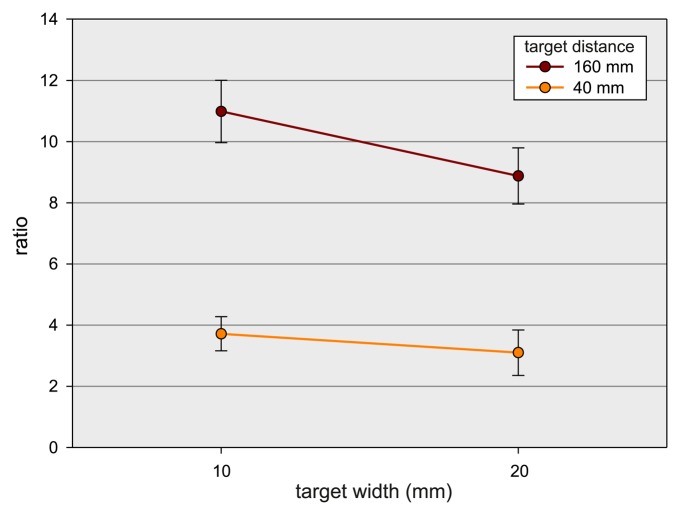
Analysis of the interaction between target distance (40 vs. 160 mm) and target width (10 *vs.* 40 mm). Trajectories were fitted to ellipses, and the ratios of the resulting radii were used as indices of circularity.

Different patterns of movement trajectories dependent on different target width/distance conditions for the LMC were observed. Figure 6 depicts accumulator plots of the movement trajectories for LMC conditions with *ID* = 1.0 (Figure 6a), *ID* = 2.3 (Figure 6b,c) and *ID* = 4.1 (Figure 6d). Trajectory space is discretized in the x-y-plane into accumulator cells, and the complete set of trajectories is mapped spatially to these cells. Each cell's value is defined by counting the intersections of movement trajectories with these cells. Afterwards, accumulator values are visualized as a heat map. It can be observed that the conditions under which the pointing gestures are performed have a high impact on the movement trajectories. Relatively large target widths result in arc-shaped trajectories (*cf.* Figure 6a,c), while this effect decreases with decreasing target widths (*cf.* Figure 6b,d). This suggests that for large target widths, the pointing gesture was incorporated into the movement to the target. For smaller target widths, the pointing movement seems to be separated from the movement to target (aiming). This separation effect is enhanced with increasing target distance. Least squares ellipse fitting [46] of the trajectory points for the aforementioned conditions were conducted to quantify the visual variations in shapes in the accumulator plots. The trajectory for each movement is fitted to an ellipse, and the ratio *r* of the resulting radii and the rotation angle *α* are analyzed. This ratio is used as an index of circularity, *i.e.*, *r* → 1 refers to a circular and increasing *r* indicates a straight, line-shaped feature. ANOVAs with the factors target distance (40 mm *vs.* 160 mm) and target width (10 mm *vs.* 40 mm) were performed for the parameters *r* and *α*. There were main effects of distance, F(1, 11) = 52.8, *p* < 0.001, $\eta_{G}^{2}=0.54$, width, F(1, 11) = 62.1, *p* < 0.001, $\eta_{G}^{2}=0.05$, as well as an interaction between both factors, F(1, 11) = 14.0, *p* < 0.01, $\eta_{G}^{2}=0.015$. Figure 7 depicts the mean values for the parameter *r*. The finding suggests that the shape is more circular for short distances than for far distances (3.41 *vs.* 9.93) and more circular for large than for small widths (6.0 *vs.* 7.35). The interaction between distance and width suggests that factor width has a stronger influence on shape at far target distances than at short distances. *Post hoc* analyses revealed that *r* significantly differed between both widths for short distances, F(1, 11) = 7.46, *p* < 0.02, $\eta_{G}^{2}=0.1$, as well as for far distances, F(1, 11) = 50.38, *p* < 0.001, $\eta_{G}^{2}=0.073$. With regard to the parameter *α*, only a significant main effect of width could be found, F(1, 11) = 15.48, *p* < 0.01, $\eta_{G}^{2}=0.14$. *α* was lower for small widths (−0.51) than for large widths (−0.2). This finding suggests that the movement trajectories were more skewed for small than for large target widths.

### 3.5. Patterns of Movement Trajectories

In sum, the shape analyses based on the ellipse fittings corroborate that the shape of the movement trajectories of the LMC depend on the difficulty of the distance/width combination of the targets. Furthermore, the shape of the trajectories indicate that the integration of the click gestures into the actual aiming movements also may depend on the target difficulty. Thus, different modes in the coordination of the hand and index finger are obviously required in dependency of the difficulty of a click and pointing movement.

## 4. Discussion and Conclusions

This study discussed the Leap Motion Controller's functionality as a pointing device in a one-dimensional Fitts' law-based test setup. With an overall *ER* of 7.2%, the error rate of the LMC is three-times higher than the error rate achieved with a standard mouse device. The *ER* of LMC movements increases more steeply with *ID* than for mouse movements. No effects of gender in the direct behavioral measures were found. Mean movement time was significantly lower with the mouse device than with the LMC (565 ms *vs.* 945 ms), and a general practice effect on *MT* was revealed. Furthermore, a general training effect was also found for throughput, and this corroborates the general practice effect found for *MT*. Interestingly, however, the effect of practice also interacted with the factors device and gender. With regard to the gender-specific training effects, training increased the throughput in female participants more strongly than in male participants. The findings for *MT* and *TP* are in contrast with the study by Rohr [31], which found lower performance in pointing movements for female than for male participants. A possible explanation for this difference may be the high level of manual dexterity needed to successfully perform the LMC pointing movement, in particular for small target widths. Further, Rohr used a more restrictive *MT* directive to stress the participant's movement-production system, which causes gender-specific movement strategies to become evident [47]. Participants were asked after the experimental session how many hours per week they were playing video games. Only three of the male participants reported playing video games (on average, 10 h per week). Two of them played role and strategic games and one of them shooter and battlefield games. Thus, because female participants performed better than male participants, it seems to be unlikely that the differences in task performance can be explained by differences in video game experience. With regard to the maximal *TP* that could be attained with the two devices, LMC clearly showed a limited performance in comparison with the mouse device. Both devices increased in *TP* with increasing *ID* levels and went into saturation at the same *ID* level. Such a finding suggests that there may be a similar basic mechanism that affects the information processing of the two devices. The bandwidth of the LMC device, however, may be reduced due to certain sources of noise. As already mentioned in the Introduction, one source of noise may be introduced by the higher number of degrees of freedom for LMC movements. Another factor of bandwidth limitation, which is also related to the degrees of freedom, may be the flicking movement of the index finger, which serves as a clicking gesture. In contrast to the pointing movements of the mouse, it seems to be a more complex movement in the coordination of the arm, hand and index finger. The higher complexity of the LMC movements is also reflected by the rating of subjective mean effort (RSME), which was generally higher for the LMC than for the mouse device. Interestingly the coordination pattern between hand and finger seems also to depend on the distance/width relations (indicated by the shape of the movement trajectories), which is not predicted by Fitts' law.

In sum, the interaction with a user interface using touch-free sensors, like the LMC, yields certain trade-offs. More degrees of freedom have to be controlled with the LMC than with a mouse device, requiring advanced motor and coordination skills. This is reflected by the overall results of the error rate, movement time and throughput. However, natural interactions by fingertip positioning and tapping offer advantages for the design of adapted user interfaces, in particular with respect to critical sterile environments. Target widths of 40mm − 20mm and target distances up to 80 mm showed comparable error rates with a standard mouse device. Hence, the LMC is suitable for specialized adapted interaction tasks, but has no ability to replace a mouse as a pointing device on a daily basis.

## 5. Limitations and Future Research

There are at least two limitations of this study that have to be considered for the comparison between mouse and LMC movements. First, both devices differ considerably in the degree of familiarity. All participants of this study are regular computer users and, thus, are highly trained in the use of the computer mouse. None of the participants, however, had interacted with an LMC device before. Nearly all studies conducted in the context of Fitts' law that have used prototypical new devices suffer from the familiarity problem. Interestingly, higher practice effects were found for the mouse than for the LMC device. However, this must not mean that the short initial training with the LMC devise was sufficient. The finding could also be interpreted in such a way that the interaction time was not long enough to gain from the additional practice. Future research with well-trained LMC users may help to clarify the familiarity problem.

A possible approach to solve the familiarity problem would be to find participants who have no experience in the use of the computer mouse. Finding such participants within the age range of the participants involved in this study seems to be virtually impossible. There is a certain chance of finding older adults with no mouse experience. However, older adults are usually slower and less accurate in the performance of aiming movement tasks as employed in the current study [48]. Therefore, it would be difficult to compare younger participants with mouse experience with older participants without mouse experience. However, comparing three groups—young adults with mouse experience, old adults without mouse experience and old adults with mouse experience—may allow one to solve the familiarity problem if it is possible to partial out the influence of age.

Second, even though the LMC device provides a similar gesture for pointing movements as the mouse device, the requirements of the motor system differ considerably. Interestingly, the *TP* by *ID* functions of both devices show a similar pattern, which may be a hint that the point and clicking movements performed with the two devices have the same underlying generalized motor programs and central pattern generators, respectively (e.g., [49]). As mentioned above, the higher degrees of freedom and the specific requirements of the coordination of finger and hand movements may introduce additional noise in the implementation of the LMC movements and, therefore, reduce *TP*.

Thus, future work will focus on the evaluation of different LMC gestures, since the pointing performance of the LMC seems to strongly depend on the choice of the pointing gesture. In this context, the performance of aiming tasks, where a selection is triggered by holding a static position for a given time slot, seems a promising alternative. The extension of the experiment to two-dimensional Fitts' law-based pointing tasks might help to further explain performance differences between devices. Furthermore, an extended model of Fitts' law for 3D aiming movements [50,51] might be utilized in a 3D pointing experiment. In this context, different selection schemes for stereoscopically-rendered scenes should be taken into account [52–54]. Furthermore, the suggestion of new Leap Motion Controller-adapted user interface designs are an interesting topic; hence, the integration of the Leap Motion Controller into current medical or industrial applications, which are also used in sterile environments, like interactive navigation tools for medical volume data (e.g., for 3D Slicer (3DSlicer, http://www.slicer.org), or Invesalius (Invesalius, http://www.cti.gov.br/invesalius/)) should be evaluated. Further, adapting gesture-controlled in-vehicle information systems (IVIS) [55] to the LMC device seams promising.

## Figures and Tables

**Table 1.** Target conditions.

**Target Distance, *D* (mm)**	**Target Width, *W* (mm)**	**Index Of Difficulty, *ID* (bits)**	***ID* Number**
40.0	40.0	1.0	1
40.0	20.0	1.6	2
80.0	40.0	1.6	2
40.0	10.0	2.3	3
80.0	20.0	2.3	3
160.0	40.0	2.3	3
80.0	10.0	3.2	4
160.0	20.0	3.2	4
160.0	10.0	4.1	5
